# Supplementary material for: Cultural & region-specific adaptation of KAP (Knowledge, attitude, and practice) tool to capture healthy lifestyle within primary care settings
Source: PLoS One. 2024 Dec 19;19(12):e0312852. doi: 10.1371/journal.pone.0312852 (PMC11658580; doi:10.1371/journal.pone.0312852)
Supplement: S1 File — (DOCX) [file pone.0312852.s001.docx]

**Knowledge, attitude, and practice towards healthy lifestyle among the primary health care corporation community: A cross-sectional survey, 2022**

# Demographic information

Now I am going to ask you some information about yourself.

| **SN** | **Question** | **Answer choices** | **Answer** |
| --- | --- | --- | --- |
| **P1Q1** | What is your age (years)? |  | ________ |
| **P1Q2** | Gender | Female | 0 |
|  |  | Male | 1 |
| **P1Q3** | Nationality |  | ________ |
| **P1Q4** | Address (Zone number) |  | ________ |
| **P1Q5** | Participant height (cm) |  | ________ |
| **P1Q6** | Participant weight (kg) |  | ________ |
| **P1Q7** | What is the highest educational level you have completed? | - Never attended school | 1 |
|  |  | - Less than high school | 4 |
|  |  | - Completed Secondary (high) school (12 classes) | 5 |
|  |  | - Trade/technical/vocational qualification | 6 |
|  |  | - University Diploma/Bachelor's degree | 7 |
|  |  | - Postgraduate degree and higher | 8 |
|  |  | - I don’t know | 77 |
|  |  | - Refused | 88 |
| **P1Q8** | What is your current employment status? | - Own business/self employed | 1 |
|  |  | - Employed for a salary | 2 |
|  |  | - Unemployed less than 1 year | 3 |
|  |  | - Unemployed 1 year or more | 4 |
|  |  | - Student | 5 |
|  |  | - Retired | 6 |
|  |  | - Labor | 7 |
|  |  | - Housewife | 8 |
|  |  | - I don’t know | 77 |
|  |  | - Refused | 88 |
| **P1Q9** | What is your marital status? | - Never married before | 1 |
|  |  | - Married | 2 |
|  |  | - Separated/Divorced/Widow | 3 |
|  |  | - I don’t know | 77 |
|  |  | - Refused | 88 |
| **P1Q10** | If Married/ Separated/ Divorced/ Widow, How many children do you have? |  | ____ |
|  |  | - I don’t know | 77 |
|  |  | - Refused | 88 |
|  |  | - Not applicable | 99 |
| **P1Q11** | During the previous year, what was the household’s average month income in QAR? | - Less than 5000 | 1 |
|  |  | - 5000-9999 | 2 |
|  |  | - 10000-19999 | 3 |
|  |  | - 20000-29999 | 4 |
|  |  | - 30000+ | 5 |
|  |  | - I don’t know | 77 |
|  |  | - Refused | 88 |
| **P1Q12** | Who do you live with? | - Alone | 1 |
|  |  | - Family | 2 |
|  |  | - Friends | 3 |
|  |  | - Work Colleagues | 4 |
|  |  | - I don’t know | 77 |
|  |  | - Refused | 88 |
| **P1Q13** | The count of people (including yourself) living in the same house (residential unit) is |  | ____ |
|  |  | - I don’t know | 77 |
|  |  | - Refused | 88 |
|  |  | - Not applicable | 99 |
| **P1Q14** | The count of bedrooms and living rooms (excluding kitchen and bath) in your residence is |  | ____ |
|  |  | - I don’t know | 77 |
|  |  | - Refused | 88 |
|  |  | - Not applicable | 99 |

# Diet

## Knowledge

I am going to ask you now some questions regarding food and your dietary habits

| **SN** | **Question** | **Answer choices** | **Answer** |
| --- | --- | --- | --- |
| **P21Q1** | What do you consider to be a healthy dietary habit for you?  *(Read each option to the respondent and ask him/her to answer yes/ no/ I don’t know. Record “1=yes” and “0=No” “77=I don’t know” in the allocated space)* | - Balanced variety of the following food categories: Vegetables, Fruit, Legumes, Cereals & Starchy Vegetables like potatoes, Milk, Dairy Products, and Fish, Poultry, Meat | ___ |
|  |  | - Eating main meals (Breakfast, lunch and/or dinner) regularly and not snacking in between | ___ |
|  |  | - Less sugar/sweet foods | ___ |
|  |  | - Eating more vegetables | ___ |
|  |  | - Eating smaller portions of any available food | ___ |
|  |  | - Using less fat in cooking | ___ |
|  |  | - Lots of fresh fruit | ___ |
|  |  | - Avoid carbohydrate rich food (Wheat, Starchy vegetables like potatoes) | ___ |
|  |  | - Lots of dairy products | ___ |
|  |  | - Lots of fresh fruit juice | ___ |
|  |  | - Avoid canned/processed food (Mortadella, Salami, Sausages, Chips) | ___ |
|  |  | - Substitute white bread with whole grain (brown) bread, in addition to eating cereals | ___ |
|  |  | - Read food labels to avoid food with high calory/ energy content. | ___ |
|  |  | - Read food labels to choose nutritious foods with high fiber content and to avoid bad fat (hydrogenated or trans-fat). | ___ |
|  |  | - Its preferrable to choose sources other than dairies as a source of calcium and vitamin D rich foods like almonds and chickpeas | ___ |
|  |  | - Choose skinless poultry and lean cuts of meat | ___ |
|  |  | - Avoid red meat | ___ |
|  |  | - It is preferrable to choose meat over legumes, nuts and seeds as a protein source | ___ |
|  |  | - Fresh fruit juice is as healthy a choice as whole fruit | ___ |
|  |  | - If you can not avoid snacks then choose unsalted nuts and seeds as part of a healthy snack | ___ |
|  |  | - Lowering salt and salty sauces consumption | ___ |
|  |  | - Keep regular hours for meals | ___ |
|  |  | - Refused | 88 |
| **P21Q2** | I will give you some examples of food. What do you think is healthy food and what is not?  *(Read each option to the respondent and ask him/her to answer Healthy/ Unhealthy/ I don’t know. Record “1=Healthy” and “2=Unhealthy” “77=I don’t know” in the allocated space)* | - Salad | ___ |
|  |  | - Any freshly prepared food which is available | ___ |
|  |  | - Deep fried vegetables | ___ |
|  |  | - Mortadella, Salami, Sausage, , pizza | ___ |
|  |  | - Sweetened soft drinks (Fanta, Cola, Pepsi …etc) | ___ |
|  |  | - Fresh whole fruits | ___ |
|  |  | - Doughnuts, cookies | ___ |
|  |  | - Jam, Desserts | ___ |
|  |  | - Chips | ___ |
|  |  | - Fresh Vegetables, Vegetable sautés, soup and curries | ___ |
|  |  | - Rice | ___ |
|  |  | - White bread/ pasta | ___ |
|  |  | - Meat/Chicken/Fish | ___ |
|  |  | - Whole grain (bread) bread | ___ |
|  |  | - Beef/chicken burgers | ___ |
|  |  | - Traditional sweets (Baklava) | ___ |
|  |  | - Refused | 88 |
| **P21Q3** | Do you know health risk associated with obesity and unhealthy food? I will mention some diseases now. Do you think that the following disease/health condition is potentially associated with obesity and unhealthy diet?  *(Read each option to the respondent and ask him/her to answer yes/no/I don’t know. Record “1=yes” and “0=No” “77=I don’t know” in the allocated space)* | - COVID19 | ___ |
|  |  | - cardiovascular diseases | ___ |
|  |  | - Cataract/Glaucoma | ___ |
|  |  | - Certain cancers | ___ |
|  |  | - Degenerative joint problems like osteoarthritis | ___ |
|  |  | - Osteoporosis | ___ |
|  |  | - Presenile dementia/Alzheimer | ___ |
|  |  | - Bad teeth | ___ |
|  |  | - Hypertension | ___ |
|  |  | - type 2 diabetes | ___ |
|  |  | - HIV/AIDS | ___ |
|  |  | - Refused | 88 |
| **P21Q4** | The term “Junk Food” from the health perspective refer to the following characteristics of food.  *(Read each option to the respondent and ask him/her to answer yes/no/I don’t know. Record “1=yes” and “0=No” “77=I don’t know” in the allocated space)* | - Food that is high in calories from sugar and/or fat, and possibly also sodium (table salt for example) | ___ |
|  |  | - Food stored for a long time | ___ |
|  |  | - Little dietary fiber, protein, vitamins, minerals or other important forms of nutritional value | ___ |
|  |  | - Bland taste food | ___ |
|  |  | - Food of low cost | ___ |
|  |  | - Refused | 88 |
| **P21Q5** | Comment on the following items as choices for a healthy lifestyle.  *(Read each option to the respondent and ask him/her to answer yes/no/I don’t know. Record “1=yes” and “0=No” “77=I don’t know” in the allocated space)* | - Maintain a healthy weight. You should aim to a body mass index between 18.5 to 25 kg/m^2^ | ___ |
|  |  | - Maintain a healthy weight. You should aim to a body mass index <30 kg/m^2^ | ___ |
|  |  | - You should always watch your waist circumference in addition to height and weight. | ___ |
|  |  | - Eat home-made food more often and explore healthy ways to prepare traditional foods | ___ |
|  |  | - Most health recommendations suggest about 4 cups water per day as sufficient. | ___ |
|  |  | - Consuming sugar sweetened drinks is an important cause of weight gain. | ___ |
|  |  | - Refused | 88 |
| **P21Q6** | I will now read out 5 foods and I would like you to tell me if they are high, medium or low in salt content  **(“Low/ Medium/ High/ I don’t know” response options for each)**  *(Read each option to the respondent and ask him/her to answer yes/no/I don’t know. Record “1=Low”, “2=Medium”, “3=High” “77=I don’t know” in the allocated space)* | - Rice | ___ |
|  |  | - Chips | ___ |
|  |  | - Ketchup | ___ |
|  |  | - Salty laban (Ayran) |  |
|  |  | - White bread | ___ |
|  |  | - Pickled vegetables | ___ |
|  |  | - Tabasco and Soy sauce | ___ |
|  |  | - Mortadella, Salami, Sausage | ___ |
|  |  | - Refused | 88 |

## Attitude

I will read you some statements. Please, give your opinion regarding those statements as “strongly agree”, “agree”, “neutral”, “disagree”, or “strongly disagree”.

*Put tick (√) in the appropriate box. The answer can be only one option for each statement.*

| **Statements** | **Strongly Agree=5** | **Agree=4** | **Neutral=3** | **Disagree=2** | **Strongly disagree=1** | **Code** |
| --- | --- | --- | --- | --- | --- | --- |
| 1. Healthy food is important |  |  |  |  |  |  |
| 1. Healthy foods are enjoyable |  |  |  |  |  |  |
| 1. I really care about what I eat |  |  |  |  |  |  |
| 1. Healthy food is not tasty |  |  |  |  |  |  |
| 1. Healthy food is for sick people |  |  |  |  |  |  |
| 1. If you do enough exercise, you can eat whatever you like |  |  |  |  |  |  |
| 1. If you don’t have any health problems, you can eat whatever you like |  |  |  |  |  |  |
| 1. I wouldn’t let my children eat junk food |  |  |  |  |  |  |
| 1. I always think of the calories in what I eat |  |  |  |  |  |  |
| 1. Soft drinks (other than fresh juice) are good in the summer |  |  |  |  |  |  |
| 1. I want to drink more pure water during the day |  |  |  |  |  |  |
| 1. I want to improve my food intake pattern |  |  |  |  |  |  |
| 1. I want to improve my family’s food pattern? |  |  |  |  |  |  |
| 1. lowering the salt in diet is important for health |  |  |  |  |  |  |
| 1. Overweight people are healthier |  |  |  |  |  |  |
| 1. Overweight people are more attractive |  |  |  |  |  |  |

## Practice

Please answer the following questions

| **SN** | **Question** | **Answer choices** | **Answer** |
| --- | --- | --- | --- |
| **P23Q1** | How often do you eat fruits (excluding juice)?  ***(Read the options to the respondent and ask him/her to choose the one correct answer)*** | - Less than once a week | 1 |
|  |  | - 1 to 3 days a week | 2 |
|  |  | - 4 to 6 days a week | 3 |
|  |  | - At least once daily | 4 |
|  |  | - More than once a day | 5 |
|  |  | - I don’t know | 77 |
|  |  | - Refused | 88 |
| **P23Q2** | How many servings of fruit (one serving = half a cup or a medium size fruit) do you eat on one of those days?  ***(Read the options to the respondent and ask him/her to choose the one correct answer)*** | - 1 | 1 |
|  |  | - 2-4 | 2 |
|  |  | - >4 | 3 |
|  |  | - I don’t know | 77 |
|  |  | - Refused | 88 |
| **P23Q3** | How often do you eat vegetables or salad (excluding juice and potatoes)?  ***(Read the options to the respondent and ask him/her to choose the one correct answer)*** | - Less than once a week | 1 |
|  |  | - 1 to 3 days a week | 2 |
|  |  | - 4 to 6 days a week | 3 |
|  |  | - At least once daily | 4 |
|  |  | - More than once a day | 5 |
|  |  | - I don’t know | 77 |
|  |  | - Refused | 88 |
| **P23Q4** | How many servings of vegetables (one serving = half a cup of solid vegetables and one cup of leafy vegetables) do you eat on one of those days?  ***(Read the options to the respondent and ask him/her to choose the one correct answer)*** | - 1-2 | 1 |
|  |  | - 3-5 | 2 |
|  |  | - >5 | 3 |
|  |  | - I don’t know | 77 |
|  |  | - Refused | 88 |
| **P23Q5** | How often do you drink natural fruit- or vegetable - juice?  ***(Read the options to the respondent and ask him/her to choose the one correct answer)*** | - Less than once a week | 1 |
|  |  | - 1 to 3 days a week | 2 |
|  |  | - 4 to 6 days a week | 3 |
|  |  | - At least once daily | 4 |
|  |  | - More than once a day | 5 |
|  |  | - I don’t know | 77 |
|  |  | - Refused | 88 |
| **P23Q6** | How many servings of natural fruit- or vegetable - juice (one serving = half a cup) do you drink on one of those days?  ***(Read the options to the respondent and ask him/her to choose the one correct answer)*** | - 1 | 1 |
|  |  | - 2-4 | 2 |
|  |  | - >4 | 3 |
|  |  | - I don’t know | 77 |
|  |  | - Refused | 88 |
| **P23Q7** | How often do you add salt or a salty sauce such as soya sauce to your food right before you eat it or as you are eating it?  ***(Read the options to the respondent and ask him/her to choose the one correct answer)*** | - Never | 1 |
|  |  | - Rarely (less than once a week) | 2 |
|  |  | - Sometimes (few times a week, but not daily) | 3 |
|  |  | - Often (At least once a day) | 4 |
|  |  | - Always (more than one meal/day) | 5 |
|  |  | - I don’t know | 77 |
|  |  | - Refused | 88 |
| **P23Q8** | How often do you eat processed food high in salt? By processed food high in salt, I mean foods that have been altered from their natural state, such as packaged salty snacks, canned salty food including pickles and preserves, salty food prepared at a fast food restaurant, cheese, smoked dried and processed meat  ***(Read the options to the respondent and ask him/her to choose the one correct answer)*** | - Never | 1 |
|  |  | - Rarely (less than once a week) | 2 |
|  |  | - Sometimes (few times a week, but not daily) | 3 |
|  |  | - Often (At least once a day) | 4 |
|  |  | - Always (more than one meal/day) | 5 |
|  |  | - I don’t know | 77 |
|  |  | - Refused | 88 |
| **P23Q12** | Have you weighed yourself in the last 6 months?  ***(Read the options to the respondent and ask him/her to choose the one correct answer)*** | - No | 0 |
|  |  | - Yes | 1 |
|  |  | - I don’t know | 77 |
|  |  | - Refused | 88 |
| **P23Q13** | What type of food do you prefer to buy usually?  ***(Read the options to the respondent and ask him/her to choose one appropriate answer)*** | - Precooked meals (Talabat/ Delivery). |  |
|  |  | - Buy ingredients and cook at home. |  |
|  |  | - Frozen readymade meals |  |
|  |  | - Canned readymade meals |  |
|  |  | - I don’t know | 77 |
|  |  | - Refused | 88 |

# Physical activity

## Knowledge

*Now I am going to ask you what you know about physical activity.*

| **SN** | **Question** | **Answer choices** | **Answer** |
| --- | --- | --- | --- |
| **P31Q1** | To lead a healthy life adults should engage in regular moderate intensity physical activities (like walking and cycling) for a minimum of ______:  ***(Read the options to the respondent and ask him/her to choose the one correct answer)*** | - At least 5 days per week (for at least 30 minutes per day) | 1 |
|  |  | - At least 5 days per week (for at least 60 minutes per day) | 2 |
|  |  | - At least 20 hours a week | 3 |
|  |  | - I don’t know | 77 |
|  |  | - Refused | 88 |
| **P31Q2** | For those preferring to engage in vigorous intensity aerobic physical activity (like running and swimming) to lead a health life it is sufficient to spend a minimum duration of ______:  ***(Read the options to the respondent and ask him/her to choose the one correct answer)*** | - At least 3 days per week (for at least 60 minutes per session). | 1 |
|  |  | - At least 3 days per week (for at least 20 minutes per session). | 2 |
|  |  | - At least 6 hours a week | 3 |
|  |  | - I don’t know | 77 |
|  |  | - Refused | 88 |
| **P31Q3** | Among the known benefits of regular physical activities:  *(Read each option to the respondent and ask him/her to answer yes/no/I don’t know. Record “1=yes” and “0=No” “77=I don’t know” in the allocated space)* | - Relieve stress | ___ |
|  |  | - Helps the digesting the food properly | ___ |
|  |  | - lower blood sugar in people with diabetes | ___ |
|  |  | - Increase the “good” cholesterol levels | ___ |
|  |  | - Increase the vitamin levels in blood | ___ |
|  |  | - Physical activity is as important as healthy food in reducing the risk of over 25 chronic health conditions | ___ |
|  |  | - Older people can protect their bones from osteoporosis by walking more frequent. | ___ |
|  |  | - Provides a good protection from COVID19 | ___ |
|  |  | - Refused | 88 |

## Attitude

I will read you some statements. Please, give your opinion regarding those statements as “strongly agree”, “agree”, “neutral”, “disagree”, or “strongly disagree”.

*Put tick (√) in the appropriate box. The answer can be only one option for each statement.*

| **Statements** | **Strongly Agree=5** | **Agree=4** | **Neutral=3** | **Disagree=2** | **Strongly disagree=1** | **Code** |
| --- | --- | --- | --- | --- | --- | --- |
| 1. Physical activity is important for maintaining a good health |  |  |  |  |  |  |
| 1. I enjoy physical activity |  |  |  |  |  |  |
| 1. Low physical activity is linked to overweight and obesity |  |  |  |  |  |  |
| 1. When I eat less I don’t need to be physically active |  |  |  |  |  |  |
| 1. I don’t want to do more physical activity because it increases my appetite and increases my weight |  |  |  |  |  |  |
| 1. TV, electronic games, smart phone and computer use should be restricted as much as possible |  |  |  |  |  |  |
| 1. I don’t need to practice more physical exercise because I don’t feel the need to loose weight |  |  |  |  |  |  |

| **SN** | **Question** | **Answer choices** | **Answer** |
| --- | --- | --- | --- |
| **P32Q9** | How important is having a normal body weight to you?  ***(Read the options to the respondent and ask him/her to choose the one correct answer)*** | - Not important at all | 1 |
|  |  | - Not important | 2 |
|  |  | - Important | 3 |
|  |  | - Very important. | 4 |
|  |  | - I don’t know | 77 |
|  |  | - Refused | 88 |
| **P32Q10** | When the weather is not so hot, and you need to reach some place which is <15 min walking distance what would you prefer to use?  ***(Read the options to the respondent and ask him/her to choose the one correct answer)*** | - Cycling | ____ |
|  |  | - Use a car / motorbike / bus | ____ |
|  |  | - Walking | ____ |
|  |  | - I will not go myself but get somebody else to do the job. | ____ |
|  |  | - Others (Specify)__________________ | ____ |
|  |  | - I don’t know | 77 |
|  |  | - Refused | 88 |
| **P32Q11** | How would you rate your physical activity status?  ***(Read the options to the respondent and ask him/her to choose the one correct answer)*** | - Physically inactive | 1 |
|  |  | - Low physical activity | 2 |
|  |  | - Fairly physically active | 3 |
|  |  | - Very physically active | 4 |
|  |  | - I don’t know | 77 |
|  |  | - Refused | 88 |
| **P32Q12** | ***For those choosing options 3 and 4 on the previous question.*** Why did you choose to be physically active?  ***(Read the options to the respondent and ask him/her to choose the one correct answer)*** | - To maintain good health | 1 |
|  |  | - My regular duty/work requires that | 2 |
|  |  | - To get or feel fit | 3 |
|  |  | - Others (specify)___________________ | 4 |
|  |  | - I don’t know | 77 |
|  |  | - Refused | 88 |
|  |  | - Not applicable | 99 |

## Practice

*Now I am going to ask about your level of practicing physical activities.*

| **SN** | **Question** | **Answer choices** | **Answer** |
| --- | --- | --- | --- |
| **P33Q1** | Do you do any vigorous-intensity physical activities at work [carrying or lifting heavy loads, digging or construction work] or sports, fitness or recreational (leisure) activities that cause large increases in breathing or heart rate like [running or football]?  ***(Read the options to the respondent and ask him/her to choose the one correct answer)*** | - No | 0 |
|  |  | - Yes | 1 |
|  |  | - I don’t know | 77 |
|  |  | - Refused | 88 |
| **P32Q2** | In a typical week, on how many days do you do vigorous intensity activities?  **(Valid values range between 1-7)** |  | ------_ |
|  |  | - I don’t know | 77 |
|  |  | - Refused | 88 |
|  |  | - Not applicable | 99 |
| **P33Q3** | How much time do you spend doing vigorous-intensity activities per day on average?  ***(Read the options to the respondent and ask him/her to choose the one correct answer)*** | - <15 min | 1 |
|  |  | - 15-29 min | 2 |
|  |  | - 30-44 min | 3 |
|  |  | - 45-59 min | 4 |
|  |  | - 1 hour + | 5 |
|  |  | - I don’t know | 77 |
|  |  | - Refused | 88 |
|  |  | - Not applicable | 99 |
| **P33Q4** | Do you do any moderate-intensity physical activities at work [brisk walking or carrying light loads] or sports, fitness or recreational (leisure) activities that cause a small increase in breathing or heart rate such as brisk walking, [cycling, swimming, volleyball]? | - No | 0 |
|  |  | - Yes | 1 |
|  |  | - I don’t know | 77 |
|  |  | - Refused | 88 |
| **P33Q5** | In a typical week, on how many days do you do moderate-intensity physical activities?  **(Valid values range between 1-7)** |  | ____ |
|  |  | - I don’t know | 77 |
|  |  | - Refused | 88 |
|  |  | - Not applicable | 99 |
| **P33Q6** | How much time do you spend doing moderate-intensity physical activities per day on average?  ***(Read the options to the respondent and ask him/her to choose the one correct answer)*** | - <15 min | 1 |
|  |  | - 15-29 min | 2 |
|  |  | - 30-44 min | 3 |
|  |  | - 45-59 min | 4 |
|  |  | - 1 hour + | 5 |
|  |  | - I don’t know | 77 |
|  |  | - Refused | 88 |
|  |  | - Not applicable | 99 |
| **P33Q7** | Do you do perform any routine low intensity physical activity such as slow-paced walking? | - No | 0 |
|  |  | - Yes | 1 |
|  |  | - I don’t know | 77 |
|  |  | - Refused | 88 |
| **P33Q8** | In a typical week, on how many days do you do this type of physical activities?  **(Valid values range between 1-7)** |  | ____ |
|  |  | - I don’t know | 77 |
|  |  | - Refused | 88 |
|  |  | - Not applicable | 99 |
| **P33Q9** | How much time do you spend doing this type of physical activities per day on average?  ***(Read the options to the respondent and ask him/her to choose the one correct answer)*** | - <15 min | 1 |
|  |  | - 15-29 min | 2 |
|  |  | - 30-44 min | 3 |
|  |  | - 45-59 min | 4 |
|  |  | - 1 hour + | 5 |
|  |  | - I don’t know | 77 |
|  |  | - Refused | 88 |
|  |  | - Not applicable | 99 |
| **P33Q10** | How many hours do you usually spend sitting or reclining on a typical day?  **(Valid values range between 0.25-24 or 77 = I don’t know)** |  | ____ |
|  |  | - I don’t know | 77 |
|  |  | - Refused | 88 |

# Smoking habit

*Now I am going to ask you questions about smoking habit.*

| **SN** | **Question** | **Answer choices** | **Answer** |
| --- | --- | --- | --- |
| **P4Q1** | Have you ever smoked any tobacco products, such as Cigarettes, cigars / pipes/ sibil/ midwakh/ khishg/ or Shisha/Argeela?  ***(Read the options to the respondent and ask him/her to choose the one correct answer)*** | - No | 0 |
|  |  | - Yes | 1 |
|  |  | - I don’t know | 77 |
|  |  | - Refused | 88 |
| **P4Q2** | Do you currently smoke any tobacco products daily?  ***(Definition: Daily means smoking at least one tobacco product every day or nearly every day over a period of a month or more)***  ***(Read the options to the respondent and ask him/her to choose the one correct answer)*** | - No | 0 |
|  |  | - Yes | 1 |
|  |  | - I don’t know | 77 |
|  |  | - Refused | 88 |
|  |  | - Not applicable | 99 |
| **P4Q3** | If currently smoking daily. How long have you been smoking in years?  ***(Valid values range between 0.1 – 70 years)*** |  | ____ |
|  |  | - I don’t know | 77 |
|  |  | - Refused | 88 |
|  |  | - Not applicable | 99 |
| **P4Q4** | If currently smoking daily. have you tried to stop smoking in the past 12 months?  ***(Read the options to the respondent and ask him/her to choose the one correct answer)*** | - No | 0 |
|  |  | - Yes | 1 |
|  |  | - I don’t know | 77 |
|  |  | - Refused | 88 |
|  |  | - Not applicable | 99 |

# Health Status

*Now I am going to ask you questions about your health.*

| **SN** | **Question** | **Answer choices** | **Answer** |
| --- | --- | --- | --- |
| **P5Q1** | Do you have a positive family history (father, mother, brother) of the following diseases?  *(Read each option to the respondent and ask him/her to answer yes/no/I don’t know. Record “1=yes” and “0=No” “77=I don’t know” in the allocated space)* | - Raised blood pressure / hypertension | ____ |
|  |  | - Diabetes | ____ |
|  |  | - Raised blood cholesterol | ____ |
|  |  | - Cardiovascular Diseases (Angina / Stroke) | ____ |
|  |  | - Asthma | ____ |
|  |  | - Chronic kidney diseases | ____ |
|  |  | - Obesity | ____ |
|  |  | - Refused | 88 |
| **P5Q2** | During the past 12 months, have you visited a doctor or other health worker who advised you of the following?  *(Read each option to the respondent and ask him/her to answer yes/no/I don’t know. Record “1=yes” and “0=No” “77=I don’t know” in the allocated space)* | - Quit smoking (using tobacco products) | ____ |
|  |  | - Reduce salt in your diet | ____ |
|  |  | - Eat at least five servings of fruit and vegetables each day | ____ |
|  |  | - Reduce fat in your diet | ____ |
|  |  | - Start or do more physical activity | ____ |
|  |  | - Maintain a healthy body weight or lose weight | ____ |
|  |  | - Refused | 88 |
|  |  | - Not applicable (I did not visit a doctor during that period) | 99 |
| **P5Q3** | Do any of your conditions or illnesses affect you in any of the following areas?  *(Read each option to the respondent and ask him/her to answer yes/no/I don’t know. Record “1=yes” and “0=No” “77=I don’t know” in the allocated space)* | - Vision (e.g. blindness or partial sight) |  |
|  |  | - Hearing (e.g. deafness or partial hearing) |  |
|  |  | - Mobility (e.g. walking short distances or climbing stairs) |  |
|  |  | - Dexterity (e.g. lifting and carrying objects, using a keyboard) |  |
|  |  | - Learning or understanding or concentrating |  |
|  |  | - Memory |  |
|  |  | - Mental health |  |
|  |  | - Stamina or breathing or fatigue |  |
|  |  | - Socially or behaviorally (e.g. associated with autism, Attention Deficit Disorder or Asperger's syndrome) |  |
|  |  | - Other (PLEASE SPECIFY) __________ |  |
|  |  | - Refused | 88 |
| **P5Q4** | I will now list some potential remedies or practices. Please rate each of them as “not effective=1”, “quite effective=2”, “very effective=3” or “I don’t know=77” in reducing the blood pressure.  ***(Read the options to the respondent and record the answer for each item)*** | - Medication | ____ |
|  |  | - Losing weight | ____ |
|  |  | - Choosing healthy diet | ____ |
|  |  | - Adopting a more physically active lifestyle | ____ |
|  |  | - Increasing the rest hours | ____ |
|  |  | - Drinking high quality spring water | ____ |
|  |  | - Refused | 88 |
|  |  | - Not applicable (I never had raised blood pressure) | 99 |
| **P5Q5** | Overall, how do you perceive your health  ***(Read the options to the respondent and ask him/her to choose the one correct answer)*** | - Very bad | 1 |
|  |  | - Bad | 2 |
|  |  | - Good | 3 |
|  |  | - Very good | 4 |
|  |  | - I don’t know | 77 |
|  |  | - Refused | 88 |

*Now I am going to ask you questions about specific chronic medical conditions.*

| **SN** | **Question**  *(Read each option to the respondent and ask him/her to answer yes/ no/ I don’t know. Record “1=yes” and “0=No” “77=I don’t know” in the allocated space)* | **Asthma (P5Q#a)** | **Cardiovascular Diseases (heart attack or angina or a stroke) (P5Q#b)** |
| --- | --- | --- | --- |
| **P5Q6** | Have you ever been told by a doctor or other health workers that you have this disease? | ______  Refused=88 | ______  Refused=88 |
| **P5Q7** | In the past two weeks, have you taken any drugs (medication) for the disease (based on a medical prescription)? | ______  Refused=88  Not applicable=99 | ______  Refused=88  Not applicable=99 |

| **SN** | **Question**  *(Read each option to the respondent and ask him/her to answer yes/ no/ I don’t know. Record “1=yes” and “0=No” “77=I don’t know” in the allocated space)* | **Raised blood pressure (P5Q#c)** | **Diabetes (P5Q#d)** | **Raised blood Cholesterol (P5Q#e)** |
| --- | --- | --- | --- | --- |
| **P5Q8** | Have you ever been told by a doctor or other health workers that you have this disease? | ______  Refused=88 | ______  Refused=88 | ______  Refused=88 |
| **P5Q9** | In the past two weeks, have you taken any drugs (medication) for the disease (based on a medical prescription)? | ______  Refused=88  Not applicable=99 | ______  Refused=88  Not applicable=99 | ______  Refused=88  Not applicable=99 |
| **P5Q10** | Have you ever had your “c=blood pressure, d=blood sugar, e=blood lipids” measured/tested by a doctor or other health worker? | ______  Refused=88 | ______  Refused=88 | ______  Refused=88 |
