# Supplementary material for: Cultural & region-specific adaptation of KAP (Knowledge, attitude, and practice) tool to capture healthy lifestyle within primary care settings
Source: PLoS One. 2024 Dec 19;19(12):e0312852. doi: 10.1371/journal.pone.0312852 (PMC11658580; doi:10.1371/journal.pone.0312852)
Supplement: Attachment — Submitted filename: PONE-D-24-12828_reviewer.pdf [file pone.0312852.s003.pdf]

PLOS ONE

Designing a KAP (Knowledge, attitude, and practice) tool to capture healthy lifestyle within primary care

--Manuscript Draft--

**Manuscript Number:**	PONE-D-24-12828
**Article Type:**	Research Article
**Full Title:**	Designing a KAP (Knowledge, attitude, and practice) tool to capture healthy lifestyle within primary care
**Short Title:**	Designing a KAP (Knowledge, attitude, and practice) tool for assessing healthy lifestyle within primary care
**Corresponding Author:**	Muslim Abbas Syed, MBBS, MPH, MSc Health Economics, MCPS HPE Ph.D<br>PHCC: Primary Health Care Corporation<br>Doha, QATAR
**Keywords:**	healthy lifestyles, primary care, knowledge attitudes and practices, non-communicable diseases
**Abstract:**	Background: Non-communicable diseases contribute to a significant global burden of disease and are associated with modifiable risk factors. These risk factors are closely related with lifestyles and eating patterns which are often culturally embedded and managed differently in various health care settings.<br>Aim of the study: To assesses the applicability and feasibility of the KAPS (Knowledge, attitude, and practice) survey in generating data about knowledge, attitudes, and practices about healthy lifestyles globally and providing foundations for testing other models or development of a newer model in this area which captures and influence behavior changes towards healthy lifestyles.<br>Methods: The KAP survey was tailored to capture the construct of healthy lifestyles (within the context of Qatar primary care settings) by reviewing existing surveys, adaptation to local context, expert consultation and feedback, pilot testing, feedback analysis, cognitive interviews and translation and validation.<br>Results: The study reports that most participants found the content comprehensive, relevant, easy to understand but considered it lengthy. The study highlights the significance of incorporating expert feedback in this exercise, acknowledging that it is an iterative process and recognizing the challenges associated with the items and domains included in such a tool.<br>Conclusion: A KAP tool specifically designed for healthy lifestyles can aid in health advocacy, monitoring modifiable risk factors, capturing rich epidemiological data to design preventive interventions, surveillance of high risks patients and strengthening the existing health information systems. Further research is needed to modify and formulate a KAP specifically designed to capture healthy lifestyles among patients which demonstrates stronger validity and generalizability.
**Order of Authors:**	Muslim Abbas Syed, MBBS, MPH, MSc Health Economics, MCPS HPE Ph.D
	Ahmed Sameer Alnuaimi, PhD
	Mohamed Ahmed Syed, PhD
	Hafiz Ahmed Mohamed
	Abduljaleel Abdullatif Zainel
**Additional Information:**	
**Question**	**Response**
**Financial Disclosure**<br><br>Enter a financial disclosure statement that describes the sources of funding for the work included in this submission. Review	The author(s) received no specific funding for this work.

Powered by Editorial Manager® and ProduXion Manager® from Aries Systems Corporation

the submission guidelines for detailed requirements. View published research articles from *PLOS ONE* for specific examples.<br><br>This statement is required for submission and **will appear in the published article** if the submission is accepted. Please make sure it is accurate.<br><br>**Funded studies**<br>Enter a statement with the following details:<br>• Initials of the authors who received each award<br>• Grant numbers awarded to each author<br>• The full name of each funder<br>• URL of each funder website<br>• Did the sponsors or funders play any role in the study design, data collection and analysis, decision to publish, or preparation of the manuscript?<br><br>Did you receive funding for this work?	
**Competing Interests**<br><br>Use the instructions below to enter a competing interest statement for this submission. On behalf of all authors, disclose any competing interests that could be perceived to bias this work—acknowledging all financial support and any other relevant financial or non-financial competing interests.<br><br>This statement is required for submission and **will appear in the published article** if the submission is accepted. Please make sure it is accurate and that any funding sources listed in your Funding Information later in the submission form are also declared in your Financial Disclosure statement.<br><br>View published research articles from *PLOS ONE* for specific examples.	The authors have declared that no competing interests exist.

**NO authors have competing interests**<br><br>Enter: *The authors have declared that no competing interests exist.*<br><br>**Authors with competing interests**<br><br>Enter competing interest details beginning with this statement:<br><br>*I have read the journal's policy and the authors of this manuscript have the following competing interests: [insert competing interests here]*<br><br>* typeset	
**Ethics Statement**<br><br>Enter an ethics statement for this submission. This statement is required if the study involved:<br><br>• Human participants<br>• Human specimens or tissue<br>• Vertebrate animals or cephalopods<br>• Vertebrate embryos or tissues<br>• Field research<br><br>Write "N/A" if the submission does not require an ethics statement.<br><br>General guidance is provided below. Consult the submission guidelines for detailed instructions. **Make sure that all information entered here is included in the Methods section of the manuscript.**	Ethics approval was received by Primary Health Care Corporation Institutional Review Board.<br>We hereby confirm that the study received a approval from PHCC IRB.<br>Verbal consent was taken from the participants.

Powered by Editorial Manager® and ProduXion Manager® from Aries Systems Corporation

**Format for specific study types**<br><br>**Human Subject Research (involving human participants and/or tissue)**<br>• Give the name of the institutional review board or ethics committee that approved the study<br>• Include the approval number and/or a statement indicating approval of this research<br>• Indicate the form of consent obtained (written/oral) or the reason that consent was not obtained (e.g. the data were analyzed anonymously)<br><br>**Animal Research (involving vertebrate animals, embryos or tissues)**<br>• Provide the name of the Institutional Animal Care and Use Committee (IACUC) or other relevant ethics board that reviewed the study protocol, and indicate whether they approved this research or granted a formal waiver of ethical approval<br>• Include an approval number if one was obtained<br>• If the study involved *non-human primates*, add *additional details* about animal welfare and steps taken to ameliorate suffering<br>• If anesthesia, euthanasia, or any kind of animal sacrifice is part of the study, include briefly which substances and/or methods were applied<br><br>**Field Research**<br><br>Include the following details if this study involves the collection of plant, animal, or other materials from a natural setting:<br>• Field permit number<br>• Name of the institution or relevant body that granted permission	
**Data Availability**<br><br>Authors are required to make all data underlying the findings described fully available, without restriction, and from the time of publication. PLOS allows rare exceptions to address legal and ethical concerns. See the PLOS Data Policy and FAQ for detailed information.	Yes - all data are fully available without restriction

A Data Availability Statement describing where the data can be found is required at submission. Your answers to this question constitute the Data Availability Statement and **will be published in the article**, if accepted.<br><br>**Important:** Stating 'data available on request from the author' is not sufficient. If your data are only available upon request, select 'No' for the first question and explain your exceptional situation in the text box.<br><br>Do the authors confirm that all data underlying the findings described in their manuscript are fully available without restriction?	
**Describe where the data may be found in full sentences. If you are copying our sample text, replace any instances of XXX with the appropriate details.**<br><br>• If the data are **held or will be held in a public repository**, include URLs, accession numbers or DOIs. If this information will only be available after acceptance, indicate this by ticking the box below. For example: *All XXX files are available from the XXX database (accession number(s) XXX, XXX.).*<br>• If the data are all contained **within the manuscript and/or Supporting Information files**, enter the following: *All relevant data are within the manuscript and its Supporting Information files.*<br>• If neither of these applies but you are able to provide **details of access elsewhere**, with or without limitations, please do so. For example:<br><br>*Data cannot be shared publicly because of [XXX]. Data are available from the XXX Institutional Data Access / Ethics Committee (contact via XXX) for researchers who meet the criteria for access to confidential data.*<br><br>*The data underlying the results presented in the study are available from (include the name of the third party*	The datasets used and/or analyzed during this study are available from the corresponding author on reasonable request.

and contact information or URL).<br>• This text is appropriate if the data are owned by a third party and authors do not have permission to share the data.<br><br>* typeset	
Additional data availability information:	Tick here if your circumstances are not covered by the questions above and you need the journal's help to make your data available.

Main document Click here to access/download;Manuscript;main Document v.1 (3) (1).docx

Title page

Designing a KAP (Knowledge, attitude, and practice) tool to capture healthy lifestyle within primary care.

Author details:

1. Dr Ahmed Sameer Alnuaimi
   PhD

Public Health Research Consultant, Clinical Affairs, Department of Clinical Research -Primary Health Care Cooperation Qatar

Doha, Qatar

Official email: asalnuaimi@phcc.gov.qa

2. Dr Muslim Abbas Syed MBChB, MPH, MSc, MCPS HPE, PhD **(Corresponding author)**

Consultant (Public Health & Health Services Research), Clinical Affairs, Department of Clinical Research -Primary Health Care Cooperation Qatar

Doha, Qatar

Official email: Musyed@phcc.gov.qa

Contact: 0097471234476

Personal email: taureanvibes@hotmail.com

3. Dr Mohamed Ahmed Syed
   PhD

Acting Director of Clinical Research, Clinical Affairs, Department of Clinical Research -Primary Health Care Cooperation Qatar

Doha, Qatar

Official email: Masyed@phcc.gov.qa

4. Dr Hafiz Ahmed Mohamed

Public Health Research Consultant, Consultant Community Medicine, Clinical Affairs, Department of Clinical Research -Primary Health Care Cooperation Qatar

Doha, Qatar

Official email: hafmohamed@phcc.gov.qa

5. Dr Abduljaleel Abdullatif Zainel

Public Health Research Consultant, Consultant Community Medicine, Clinical Affairs, Department of Clinical Research -Primary Health Care Cooperation Qatar

Doha, Qatar

Official email: azainel@phcc.gov.qa

Abstract

Background: Non-communicable diseases contribute to a significant global burden of disease and are associated with modifiable risk factors. These risk factors are closely related with lifestyles and eating patterns which are often culturally embedded and managed differently in various health care settings.

Aim of the study: To assesses the applicability and feasibility of the KAPS (Knowledge, attitude, and practice) survey in generating data about knowledge, attitudes, and practices about healthy lifestyles globally and providing foundations for testing other models or development of a newer model in this area which captures and influence behavior changes towards healthy lifestyles.

Methods: The KAP survey was tailored to capture the construct of healthy lifestyles (within the context of Qatar primary care settings) by reviewing existing surveys, adaptation to local context, expert consultation and feedback, pilot testing, feedback analysis, cognitive interviews and translation and validation.

Results: The study reports that most participants found the content comprehensive, relevant, easy to understand but considered it lengthy. The study highlights the significance of incorporating expert feedback in this exercise, acknowledging that it is an iterative process and recognizing the challenges associated with the items and domains included in such a tool.

Conclusion: A KAP tool specifically designed for healthy lifestyles can aid in health advocacy, monitoring modifiable risk factors, capturing rich epidemiological data to design preventive interventions, surveillance of high risks patients and strengthening the existing health information systems. Further research is needed to modify and formulate a KAP specifically designed to capture healthy lifestyles among patients which demonstrates stronger validity and generalizability.

Introduction:

The World Health Organization recognizes 'health' as a multifaceted concept(1). There are multiple factors influencing the occurrence of disease and its associated health implications with affect both the individual patients and the related communities(2, 3). In recent literature there is increased emphasizes on the significance of the wider determinants of health particularly understanding the socio-cultural and economic aspects in which healthcare interventions are implemented(4, 5). Various tools have been designed and utilized to capture such information by conducting cross sectional surveys. The most widely used and known tool in this regard is the knowledge, attitude, and practice (KAP) survey.(6-8)

The KAP survey at its inception was utilized in family planning and population studies(9). The tool was applied to capture information on knowledge, attitudes, and practices in family planning that could further direct program initiatives across the globe. The KAP survey over the last few decades has established as one of the most common methodologies to investigate health behavior and to extract information on health seeking practices. However, despite its widespread application there is critique within the scientific committee pertaining to the applicability of the tool in various geographical settings and the accuracy of the data provided about knowledge, attitudes, and practices which can be utilized for purposes of planning of health program(10). Moreover, there are other models that may be considered when a program is designed to fill the gap in knowledge pertaining to specific conditions leading to behavioral changes and favorable associated outcomes. These models include Behavioral Learning Theory, Health Belief Model, Social Cognitive Theory, Theory of Reasoned Action or Theory of Planned Behavior, and Ecological and Social Ecological Models.(11)

Non communicable diseases are a major public health concern worldwide and contributes to a significant portion of burden of disease. Alarmingly, Qatar has one of the highest prevalence of diabetes worldwide and a high proportion of people visiting non-communicable disease (NCD) clinics in primary care settings have diabetes(12, 13)

There are various modifiable risk factors associated with non-communicable diseases such as poor diet, physical inactivity or sedentary lifestyles and addiction such as tobacco

consumption(14, 15). These modifiable risk factors can be controlled through healthy lifestyle practices and can prevent the occurrence of non-communicable diseases and reduce the overall prevalence(16, 17). Therefore, it is important to define the construct of healthy lifestyles and its significance towards preventing or reducing the prevalence of non-communicable disease within Qatar and its primary health care settings. Furthermore, literature substantiates the fact that healthy lifestyles is an important construct and needs to be captured utilizing models which need to be tailored considering the diverse socio-cultural and economic factors within different geographical settings that can influence it.(18, 19)

To plan an intervention at the national level using primary health care data a multiphase project was set into motion. The first part assessed the magnitude of NCD problem and the non-modifiable risk factors. The current study is the second phase of the project exploring the modifiable risk factor. The study aimed to utilize and modify KAP survey within Qatar primary settings to investigate the level of awareness of primary health care population about the concept of healthy diet and active lifestyles. The study is the first of its kind which assesses the applicability and feasibility of the KAPS survey in generating data about knowledge, attitudes and practices about healthy lifestyles and providing foundations for testing other models or development of a newer model in this area which can capture and influence behavior changes towards healthy lifestyles and desired health outcomes.

Methods

The KAP survey was tailored to capture the construct of healthy lifestyles (within the context of Qatar primary care settings) including the following steps:

1. **Review Existing Surveys:** The first step included review of existing KAP surveys related to healthy lifestyles and NCD prevention to identify relevant questions and items to outline the foundations of the survey. A pool of relevant items and domains encompassing knowledge, attitude and practices components were mainly derived from the WHO STEPwise survey instrument(20) and other KAP surveys (utilized in other studies pertaining to non-communicable diseases and its associated risk factors).(21, 22)

2. **Adaptation to Local Context:** The draft version of the modified KAP tool was verified and amended to comply with Qatar Dietary Guidelines(23) to assure that the tool is culturally suitable for the local population.

3. **Expert Consultation and feedback:** The first draft of the modified tool was shared by a panel of 13 experts which mainly included two Community Medicine Consultant Professors, two Community Medicine Consultants with research background, one community and lifestyle medicine expert, five health couches (dietitians), two community medicine specialists and one public health expert from 25/02/2023 to 14/03/2023. Verbal consent was obtained from the participants to engage in this exercise. The participants were given detailed information about the purpose of the research and the significance of their feedback in context to promoting healthy lifestyles and preventing non-communicable diseases and were given the opportunity to ask any questions. The participants were then asked directly about their willingness to participate in the study. This step was undertaken to ensure the content validity(24) of the tool. For each questionnaire item, the panelist indicated whether the specific knowledge/ Attitude/ Practice item measured is essential, useful but not essential, or not necessary as a contributor to the overall scale measure of that construct. In addition, the panelist indicated the reason for unfavorable (not essential) rating and writes any suggestion to improve the phrasing of the question. The reasons for unfavorable opinion on the side of the panelist was coded as 1=irrelevant to the construct tested, 2=Too technical and difficult to the audience, 3=Repeated elsewhere and 4=others (specify). The study tool underwent iterative amendments and improvements based on the structured panelist review feedback. The content validity ratio (CVR) for each item was computed. To determine whether an item is essential, its minimum value was compared with minimum values that depend on the number of experts who contributed ratings (a value of 0.62 for a panel of 10 experts)(25). Analysis of CVR values of the original version of the questionnaire form showed that more than 50% of questions had a CVR value of 1 (complete agreement). Another 26% had a value of 0.85 and 16.5% had a CVR of 0.69. Smaller CVR values were obtained in 4 questions only (around 5%).

$$\text{CVR}_i = \frac{n_e - \frac{N}{2}}{\frac{N}{2}},$$

Where:

CVR_i = value for an item on the test

n_e = number of experts indicating that an item is essential

N = total number of experts in the panel

4. **Considering cultural sensitivity:** Moreover, the panel also provided feedback regarding the various items to ensure that the survey considered cultural sensitivities and religious beliefs specific to Qatar to eliminate or rephrase any items that might be considered too technical, insensitive, or offensive.

5. **Pilot Testing:** The next step involved administering the initial draft of the survey to a small sample (n=11) of primary care patients attending healthcare facilities in Qatar from 25/04/2023 to 05/05/2023. Verbal consent was taken from services users before participating in the survey. The participants were given detailed information about the purpose of the research and the significance of their feedback and were given the opportunity to ask any questions. The participants were then asked directly about their willingness to participate in the study. Their feedback on strengthened the face validity(26) of the instrument mainly relating to question clarity, comprehension, and relevance.

6. **Feedback Analysis:** The findings of the pilot testing were utilized to analyze and determine whether there were any problematic questions, confusing wording, or items that were not culturally relevant in the modified KAP tool.

7. **Further modification and Refinement:** Based on the feedback analysis, necessary modifications were made to the survey items.

8. **Cognitive Interviews:** Furthermore, cognitive interviews were conducted with a subset of participants to explore how they interpreted and responded to the modified survey items. This step was included to further identify any underlying misconceptions or confusion.

9. **Finalization of the tool:** The feedback from both pilot testing rounds, and cognitive interviews was consolidated to make final adjustments to the survey items, ensuring they accurately captured participants' knowledge, attitudes, and practices related to healthy lifestyles and NCD prevention.

10. **Translation and Validation:** The survey was translated into Arabic to maintain the accuracy of the content. A validation process (backward translation) was conducted to ensure the translated version maintained the intended meaning.

Results:

Demographic details of the expert panel

The demographic details of the expert panel involved in grading each questionnaire item (for each questionnaire item) for the initial modified KAP tool for healthy lifestyles is demonstrated in table 1. A significant percentage include dieticians as shown in table 1. The work experience of dieticians ranged between 2 to 5 years, whereas community medicine consultant professor and public health expert had more than 10 years of experience (table 1).

Table 1: Frequency distribution of panelists by specialty.

Job title	Years of experience	N	%
Health coaches (dietitians)	Between 2 to 5 years	5	38.5
Community Medicine Consultant Professors	More than 10 years	2	15.4
Community Medicine Consultant with research background	Between 5-10 years	2	15.4
Community medicine specialists	Between 5-10 years	2	15.4
Community and lifestyle medicine expert	Between 5-10 years	1	7.7
Public health expert	More than 10 years	1	7.7
**Total**		**13**	**100**

Content validity ratio values (CVR)

Analysis of grading of the 73 questionnaire items (complete questionnaire included as supplementary document) included by the panel of experts (n=13) demonstrated that more than half of questions (52.1%) have a CVR value of which is followed by 26% with a value of 0.85 as depicted in table 2.

Table 2: Frequency distribution of the CVR (Content Validity Ratio) of the 73 questionnaire items.

CVR Value	N	%
1	38	52.1
0.7-0.92	19	26
0.62-0.69	12	16.4
<0.62	4	5.5
**Total**	**73**	**100**

Service users' (n=11) responses of the KAP feasibility survey

Most of the participants (n=8) took 45-60 minutes to complete the survey and responded that the questions were clear and easy to understand (91%), were relevant to and covered important domains of healthy diet and lifestyles, physical activity and associated attitudes as illustrated in table 3 and 4.

Table 3: Describing the results of KAP feasibility survey on a sample of 11 service users: Anticipated duration of completing the questionnaire.

	N	%
Approximately how long did it take you to complete the questionnaire?		
15 minutes	0	0.0
30 minutes	3	27.3
45 minutes	2	18.2
60 minutes	6	54.5
Total	11	100.0
What is your opinion of the length of the questionnaire in assessing healthy eating and active lifestyle?		
Too long	9	81.8
Too short	0	0.0
Appropriate	2	18.2

Total	11	100.0

Table 4: Describing the results of KAP feasibility survey on a sample of 11 service users: general feedback items.

Positive feedback (Total N=11)	N	%
The questions were clear	10	90.9
Found the wording of some questions difficult to understand?	3	27.3
Felt that the questionnaire omits issues considered to be important to investigate	0	0.0
Had difficulties completing the questionnaires	0	0.0
Had additional comments or suggestions for improvement	0	0.0

Describing the results of KAP feasibility survey on a sample of 11 service users: relevance of healthy diet section.

Positive feedback (Total N=11)	N	%
Thought that the questions about the Healthy Diet knowledge section are relevant to measuring knowledge about healthy food and eating?	10	90.9
Thought that the questions about the Healthy Diet attitudes section are relevant to measuring attitudes towards healthy food and eating?	11	100.0
Thought that the questions about the Healthy Diet practice section are relevant to measuring healthy food and eating behaviors?	11	100.0

Describing the results of KAP feasibility survey on a sample of 11 service users: relevance of physical activity section.

Positive feedback (Total N=11)	N	%
Thought that the questions about the Physical activity knowledge section are relevant to measuring knowledge about the role of active lifestyle and healthy weight?	11	100.0
Thought that the questions about the Physical activity attitude section are relevant to measuring attitudes towards active lifestyle and healthy weight?	11	100.0
Thought that the questions about the Physical activity practice section are relevant to measuring the physical activity practice?	11	100.0

Discussion:

The key findings of the study provide empirical evidence and application of standardized steps (based on theory and practice) pertaining to tailoring existing tools and surveys to formulate a KAP questionnaire to capture healthy lifestyles in a specific regional and cultural settings. The

study highlights the significance of incorporating expert feedback in this exercise, acknowledging that it is an iterative process and recognizing the challenges associated with the items and domains included in such a tool. The study reports that most participants found the content comprehensive, relevant, easy to understand but considered it lengthy.

Evidence suggests that while designing a KAP tool one of the essential principles is to consider the completeness of the questionnaire that it comprehensively captures the knowledge, attitudes and practices relating to the specific key research topic being investigated(27-29). There are challenges associated with that due to the time constrains within healthcare settings. The healthcare providers are expected to deliver healthcare services within defined time frames which is further exacerbated with additional workload and unexpected patient influx particularly in emergency departments(30-32). The administration of such a tool can be perceived as an additional task for healthcare providers and patients which makes the length of the questionnaire a key issue. Moreover, healthy lifestyles are a multi-faceted and complex concept and can vary significantly within different geographical settings based on cultural and sociodemographic factors. Our study attempted to tailor the tool according to Qatar dietary guidelines. It is important to consider regional dietary patterns when adopting similar strategies to design such a tool for a specific setting.

As previously mentioned, non-communicable diseases contribute to a significant burden of disease globally and have economic implications due to the high costs associated with managing these disease conditions which are chronic in nature and have adverse health outcomes including a poor quality of life and disability (12, 13). Application of a KAP tool specifically designed for healthy lifestyles can aid in health advocacy, monitoring the modifiable risk factors, capturing rich epidemiological data to design preventive interventions, surveillance of high risks patients and strengthening the existing health information systems.

The key strengths of the study include that it adhered to the key theoretical (evidence based) steps mentioned in literature to design the tool. Feedback was received by panel of experts pertaining to the inclusion of the relevant domains and items within the tool while considering the cultural and religious sensitives. It was ensured to eliminate or rephrase any items that might

be considered too technical, insensitive, or offensive content validity and the relevance of the tool for the cohort of participants it is designed for. Moreover, the findings of the pilot testing were utilized to analyze and determine whether there were any problematic questions, confusing wording, or items that were not culturally relevant in the modified KAP tool. One of the limitations of the study is that the tool is region specific and cannot be applied in non-eastern Mediterranean settings. However, this limitation is due to complying to the key principle while designing such a tool that it is modified in accordance with the target population and healthcare systems understanding and preferences towards knowledge, attitudes, and practices to healthy lifestyles.

Conclusion

A KAP tool specifically designed for healthy lifestyles have a broad range of benefits for patients and lead to desired health outcomes. Such a tool can aid in health advocacy, monitoring modifiable risk factors, capturing rich epidemiological data to design preventive interventions, surveillance of high risks patients and strengthening the existing health information systems. Further research is needed to modify and formulate a KAP specifically designed to capture healthy lifestyles among patients which demonstrates stronger validity and generalizability.

Key messages from the study:

- A KAP tool designed to capture healthy lifestyles should take into consideration incorporating the regional and cultural determinants that might influence its applicability for a specific geographical healthcare setting.
- Designing a KAP tool for capturing healthy lifestyles is an iterative process which should be substantiated with expert and pilot testing among potential patients.
- A KAP tool for capturing healthy lifestyles should be comprehensive encompassing the necessary items and domains while acknowledging the limitations such as the feasibility and its applicability within a healthcare setting.
- A KAP tool specifically designed for healthy lifestyles can aid in health advocacy, monitoring the modifiable risk factors, capturing rich epidemiological data to design preventive interventions, surveillance of high risks patients and strengthening the existing health information systems.
- Further research is needed to modify and formulate a KAP specifically for healthy lifestyles with stronger validity and generalizability within different healthcare settings

Abbreviations

KAP (Knowledge, attitude, and practice)

NCDs (non-communicable diseases)

References

1. Kickbusch I. The contribution of the World Health Organization to a new public health and health promotion. American journal of public health. 2003;93(3):383-8.
2. Ma L, Tang Z, Zhang L, Sun F, Li Y, Chan P. Prevalence of frailty and associated factors in the community-dwelling population of China. Journal of the American Geriatrics Society. 2018;66(3):559-64.
3. Van Zyl S, Van Rooyen FC, Joubert G, Kruger WH, Walsh CM. A Comparison of the Socio-Behavioral-Metabolic Risk Profiles and Associated Factors for Chronic Diseases of Lifestyle in Urban and Rural Communities in Central South Africa. Frontiers in Public Health. 2020;8:570676.

4. Bambra C, Gibson M, Sowden A, Wright K, Whitehead M, Petticrew M. Tackling the wider social determinants of health and health inequalities: evidence from systematic reviews. Journal of Epidemiology & Community Health. 2010;64(4):284-91.
5. Nobles J, Christensen A, Butler M, Radley D, Pickering K, Saunders J, et al. Understanding how local authorities in England address obesity: A wider determinants of health perspective. Health Policy. 2019;123(10):998-1003.
6. Launiala A. How much can a KAP survey tell us about people's knowledge, attitudes and practices? Some observations from medical anthropology research on malaria in pregnancy in Malawi. Anthropology Matters. 2009;11(1).
7. AshaRani P, Abdin E, Kumarasan R, Kumar FDS, Shafie S, Jeyagurunathan A, et al. Study protocol for a nationwide Knowledge, Attitudes and Practices (KAP) survey on diabetes in Singapore's general population. BMJ open. 2020;10(6):e037125.
8. El-Kholei AO, Yassein GA. Embedding sustainability and SDGs in architectural and planning education: reflections from a KAP survey, Egypt. Archnet-IJAR: International Journal of Architectural Research. 2023.
9. Cleland J. A critique of KAP studies and some suggestions for their improvement. Studies in Family Planning. 1973;4(2):42-7.
10. Hauser P. The limitations of KAP surveys. Social research in developing countries. 1993:65-9.
11. Baranowski T, Cullen KW, Nicklas T, Thompson D, Baranowski J. Are current health behavioral change models helpful in guiding prevention of weight gain efforts? Obes Res. 2003;11 Suppl:23s-43s.
12. Jaam M, Ibrahim MIM, Kheir N, Hadi MA, Diab MI, Awaisu A. Assessing prevalence of and barriers to medication adherence in patients with uncontrolled diabetes attending primary healthcare clinics in Qatar. Primary care diabetes. 2018;12(2):116-25.
13. Zidan A, Awaisu A, El-Hajj MS, Al-Abdulla SA, Figueroa DCR, Kheir N. Medication-related burden among patients with chronic disease conditions: perspectives of patients attending non-communicable disease clinics in a primary healthcare setting in Qatar. Pharmacy. 2018;6(3):85.
14. Adams ML, Grandpre J, Katz DL, Shenson D. The impact of key modifiable risk factors on leading chronic conditions. Preventive medicine. 2019;120:113-8.
15. Leventhal AM, Huh J, Dunton GF. Clustering of modifiable biobehavioral risk factors for chronic disease in US adults: a latent class analysis. Perspectives in Public Health. 2014;134(6):331-8.
16. Gill DP, Blunt W, Boa Sorte Silva N, Stiller-Moldovan C, Zou G, Petrella R. The Health e Steps™ lifestyle prescription program to improve physical activity and modifiable risk factors for chronic disease: A pragmatic randomized controlled trial. BMC Public Health. 2019;19:1-15.
17. Brodie A, Pavey T, Newton C, Sendall MC. Australian bus drivers' modifiable and contextual risk factors for chronic disease: A workplace study. Plos one. 2021;16(7):e0255225.
18. Loef M, Walach H. The combined effects of healthy lifestyle behaviors on all cause mortality: a systematic review and meta-analysis. Preventive medicine. 2012;55(3):163-70.
19. Cockerham WC. Health lifestyle theory and the convergence of agency and structure. Journal of health and social behavior. 2005;46(1):51-67.
20. World Health Organization (WHO). Standard STEPS instrument 2020 [Available from: https://www.who.int/publications/m/item/standard-steps-instrument.
21. Demaio AR, Dugee O, Amgalan G, Maximenco E, Munkhtaivan A, Graeser S, et al. Protocol for a national, mixed-methods knowledge, attitudes and practices survey on non-communicable diseases. BMC Public Health. 2011;11(1):961.
22. Oli N, Vaidya A, Pahkala K, Eiben G, Krettek A. Knowledge, attitude and practice on diet and physical activity among mothers with young children in the Jhaukhel-Duwakot Health Demographic Surveillance Site, Nepal. PLoS One. 2018;13(7):e0200329.

23. The Ministry of Public Health HPaN-cDPHD. Qatar Dietary Guidelines. Doha, Qatar: Ministry of Public Health; 2015.
24. Beckstead JW. Content validity is naught. International journal of nursing studies. 2009;46(9):1274-83.
25. Miller LA, Lovler RL. Foundations of psychological testing: A practical approach: Sage publications; 2018.
26. Holden RR. Face validity. The corsini encyclopedia of psychology. 2010:1-2.
27. Jimam NS, Ismail NE, Dapar ML. Development and validation of instrument for assessing patients' knowledge, attitude, and practice (KAP) on uncomplicated malaria management. Journal of Applied Pharmaceutical Science. 2019;9(6):033-42.
28. Dayal B. Development of an e-health tool for young adults at risk of cardiovascular disease based on their kap score. Annals of Horticulture. 2018;11(2):175-82.
29. Elsahoryi NA, Altamimi E, Subih HS, Hammad FJ, Woodside JV. Educational intervention improved parental Knowledge, Attitudes, and Practices (KAP) and adherence of patients with celiac disease to gluten-free diet. International journal of food science. 2020;2020.
30. Heiden M, Widar L, Wiitavaara B, Boman E. Telework in academia: associations with health and well-being among staff. Higher Education. 2021;81:707-22.
31. Mohanty A, Kabi A, Mohanty AP. Health problems in healthcare workers: A review. Journal of family medicine and primary care. 2019;8(8):2568.
32. Cramer RJ, Ireland JL, Hartley V, Long MM, Ireland CA, Wilkins T. Coping, mental health, and subjective well-being among mental health staff working in secure forensic psychiatric settings: Results from a workplace health assessment. Psychological services. 2020;17(2):160.

Questionnaire

Click here to access/download
Supporting Information
KAP healthy Lifestyles PHCC Questionnaire-English-7.docx

Responses to reviewer comments:

(1) Please upload a copy of the original ethics approval letter from Primary Health Care Corporation Institutional Review Board, as required by question 1 of the human subject research checklist. If the original letter is not in English, please also provide an English translated version in the supporting file.

Reply: Thank you for the comment and highlighting this. We can confirm that the original ethics approval is now attached with submission. The letter is in English.

(2)Please report in the Methods section the start and end date of the recruitment period for this study in DD/MM/YYYY format.

Reply: Thank you for the comment. We have made the necessary changes as advised above in the revised submission.

(3)Please provide additional details regarding participant consent. In the ethics statement in the Methods, you have specified that verbal consent was obtained. Please provide additional details regarding how this consent was documented and witnessed, and state whether this was approved by the IRB.

Reply: Thank you for the important. Details of the verbal consent are now mentioned in the methods section, and this was approved by IRB as rightly highlighted in the query.

(4)PLOS ONE has specific requirements for studies that are presenting a new method or tool as the primary focus, including a newly developed or modified questionnaire or scale (https://journals.plos.org/plosone/s/submission-guidelines#loc-methods-software-databases-and-tools.) One requirement is that the questionnaire or scale must be openly available under a license no more restrictive than CC BY. In light of this, before we proceed, please include a copy of your questionnaire or scale as a Supporting Information file or provide a link if it is available through an online repository. By uploading a copy of the questionnaire you have used for this study, you are confirming that you have obtained all the necessary permissions to publish the questionnaire CC BY and have included all proper attributions in your manuscript. For more detail, please see here: https://journals.plos.org/plosone/s/licenses-and-copyright. If you are unable to obtain the necessary permissions, please contact us so that we can withdraw your manuscript from consideration."

Reply: Thank you for the important suggestion. The questionnaire is now attached as supplementary document in the revised submission.
