# Supplementary material for: Cultural & region-specific adaptation of KAP (Knowledge, attitude, and practice) tool to capture healthy lifestyle within primary care settings
Source: PLoS One. 2024 Dec 19;19(12):e0312852. doi: 10.1371/journal.pone.0312852 (PMC11658580; doi:10.1371/journal.pone.0312852)
Supplement: Attachment — Submitted filename: response to reviewers.docx [file pone.0312852.s002.docx]

**Responses to reviewer comments:**

(1) Please upload a copy of the original ethics approval letter from Primary Health Care Corporation Institutional Review Board, as required by question 1 of the human subject research checklist. If the original letter is not in English, please also provide an English translated version in the supporting file.

Reply: Thank you for the comment and highlighting this. We can confirm that the original ethics approval is now attached with submission. The letter is in English.

(2)Please report in the Methods section the start and end date of the recruitment period for this study in DD/MM/YYYY format.

Reply: Thank you for the comment. We have made the necessary changes as advised above in the revised submission.

(3)Please provide additional details regarding participant consent. In the ethics statement in the Methods, you have specified that verbal consent was obtained. Please provide additional details regarding how this consent was documented and witnessed, and state whether this was approved by the IRB.

Reply: Thank you for the important. Details of the verbal consent are now mentioned in the methods section, and this was approved by IRB as rightly highlighted in the query.

(4)PLOS ONE has specific requirements for studies that are presenting a new method or tool as the primary focus, including a newly developed or modified questionnaire or scale (<https://journals.plos.org/plosone/s/submission-guidelines#loc-methods-software-databases-and-tools>.) One requirement is that the questionnaire or scale must be openly available under a license no more restrictive than CC BY. In light of this, before we proceed, please include a copy of your questionnaire or scale as a Supporting Information file or provide a link if it is available through an online repository. By uploading a copy of the questionnaire you have used for this study, you are confirming that you have obtained all the necessary permissions to publish the questionnaire CC BY and have included all proper attributions in your manuscript. For more detail, please see here: <https://journals.plos.org/plosone/s/licenses-and-copyright>. If you are unable to obtain the necessary permissions, please contact us so that we can withdraw your manuscript from consideration."

Reply: Thank you for the important suggestion. The questionnaire is now attached as supplementary document in the revised submission.
