# Supplementary material for: Cultural & region-specific adaptation of KAP (Knowledge, attitude, and practice) tool to capture healthy lifestyle within primary care settings
Source: PLoS One. 2024 Dec 19;19(12):e0312852. doi: 10.1371/journal.pone.0312852 (PMC11658580; doi:10.1371/journal.pone.0312852)
Supplement: Attachment — Submitted filename: response to reviewer comments.docx [file pone.0312852.s004.docx]

Dated: (16/07/2024)

Dear Editor,

We would like to thank you and the reviewers for the invaluable comments and feedback on our submitted manuscript, " Designing a KAP (Knowledge, attitude, and practice) tool to capture healthy lifestyle within primary care". We appreciate the time and effort that you and the reviewers have invested in meticulously evaluating our work and the attention to detail to the various components of the study.

We have carefully reviewed the feedback and made a thorough effort to address all the reviewers' comments, revise the manuscript and make the necessary changes accordingly. We believe these revisions have strengthened the manuscript and addressed most of the reviewers' concerns.

We have now submitted a revised manuscript with tracked changes for your review as instructed. We hope that the revisions meet with your approval and look forward to any further feedback you may have.

Thank you again for your kind support.

Sincere Regards

Dr Syed M Abbas

MBChB MPH MSc MRCGP MCPS HPE MS PhD

**Reviewer #1:** The presents study is conducted to assess KAP (Knowledge, attitude, and practice) tool to capture healthy lifestyle within primary care, the study is done one the indigenous sample of Qatar and may benefit from the following suggestions

Abstract
• Background: Briefly mention specific examples of modifiable risk factors (e.g., diet, physical activity)

**Response: Thank you for the comment. We have now mentioned some examples of modifiable risk factors in the background section as kindly suggested.**

• Aim: Instead of "globally," specify the target population (e.g., in a specific region or cultural context).

**Response: Thank you for the important suggestion. We have now changed ‘globally’ to eastern Mediterranean settings which is more specific to study as kindly highlighted in your comment.**

• Conclusion: Emphasize the broader impact beyond modifiable risk factors. Can the KAPS contribute to designing interventions to improve health outcomes?
Consider shortening sentences to improve readability, especially in the background and methods sections.

**Response: Thank you for highlighting an important aspect of significance of KAP tool beyond modifiable risk factors. The broader aspects are now highlighted in the conclusion (marked in red). The suggestion of shortening sentences has also been carefully considered in the text of the manuscript.**

Introduction
• Shorten the opening about the multifaceted nature of health. Briefly state that various factors influence health outcomes for individuals and communities.

**Response: Thank you for your kind suggestion. It was insightful suggestion from your end and is much appreciated. It improves the readability and further strengthens the introduction component. The opening of multifaceted nature of health is now shortened by deleting a sentence as highlighted in track changes in the revised main manuscript. The diverse range of social, economic, and environmental factors are now mentioned within the wider determinants of health as kindly suggested.**

• Combine similar points. Merge the sections about the importance of socio-economic factors and the critique of the KAPS tool.

**Response: Thank you for the important suggestion. The paragraph pertaining to the importance of socio-economic factors and the critique of the KAPS tools is merged as kindly suggested.**

• Highlight the gap your research addresses. Emphasize the lack of research on the KAPS tool's applicability in capturing healthy lifestyle knowledge in Qatar's primary care setting.

**Response: Thank you for highlighting an important point. This has been incorporated in the introduction section highlighted in track changes.**

• Cite sources directly related to Qatar and NCDs. Focus on references that highlight the prevalence of NCDs and modifiable risk factors in Qatar.

**Response: Thank you for highlighting an important point. The references have been added which refer to NCDs, Modifiable risk factors in Qatar. This is highlighted in track changes.**

• State your research question clearly. Briefly state what you aim to achieve by assessing the KAPS tool in this context.

**Response: Thank you for the important suggestion. The research question of the study is now clearly highlighted in the last paragraph as kindly advised. As suggested, we have now mentioned that the findings of the methodology study provide foundations for testing other models or development of a newer model in this area which can capture and influence behavior changes towards healthy lifestyles and desired health outcomes within primary care settings in the state of Qatar.**

Methods

• Sample Size: While you mention pilot testing with 11 participants, a larger sample size might be recommended to strengthen the generalizability of the pilot testing findings. What is the reference for it?

**Response: Thank you for the insightful comment. We truly appreciate the attention to detail from your end to further improve the study. The references are as below and added in text thanks to your kind suggestion:**

**a. Andrade C, Menon V, Ameen S, Kumar Praharaj S. Designing and Conducting Knowledge, Attitude, and Practice Surveys in Psychiatry: Practical Guidance. Indian J Psychol Med. 2020;42(5):478-481. Published 2020 Aug 27. doi:10.1177/0253717620946111.**

**b. Guidelines for Assessing Nutrition-related Knowledge, Attitudes and Practices. Rome: Food and Agriculture; 2014 (http://www.fao.org/3/i3545e/i3545e05.pdf, accessed on 07/07/2024)**

• Description of Pilot Testing and Cognitive Interviews: Consider elaborating on the format of the pilot testing and cognitive interviews (e.g., individual interviews, focus groups).

**Response: Thank you for highlighting an important point to strengthen the methods section. We have now added details on pilot testing and cognitive interviews as kindly suggested. Changes are highlighted in track changes.**

**‘The interviews were conducted individually among participants. This step was included to further identify any underlying misconceptions or confusion.** **The cognitive interview data was analyzed by synthesis and reduction(1, 2). This involved the following steps i) the initial step of conducting cognitive interviews to generate interview text (collecting narratives from respondents) ii) synthesizing detailed summaries of interview text iii) producing thematic schema by comparing summarizes across respondents iv) constructing advanced schema by comparing advanced themes across subgroups and v) drawing conclusions’. (3)**

• Demographic characteristics of the participants should be added

**Response: Thank you for the important suggestion. The demographic details of the panelists are now added in table 1 as kindly suggested (highlighted in track changes).**

• Data Analysis for Pilot Testing and Cognitive Interviews: mention how you analyzed the feedback from the pilot testing and cognitive interviews.

**Response: Thank you for the insightful comment. As kindly suggested details on analysis of the cognitive interviews are now added. The changes are highlighted as track changes in the main document. The results of pilot testing are depicted in table 4 as frequency and distribution (highlighted in track changes).**

**‘The cognitive interview data was analyzed by synthesis and reduction(1, 2). This involved the following steps i) the initial step of conducting cognitive interviews to generate interview text (collecting narratives from respondents) ii) synthesizing detailed summaries of interview text iii) producing thematic schema by comparing summarizes across respondents iv) constructing advanced schema by comparing advanced themes across subgroups and v) drawing conclusions’. (3)**

**The responses to the open ended question were analysed using framework thematic analysis.(4) This approach encompasses ‘interpreting, exploring, and reporting patterns and clusters of meaning within the given data’(5) and was facilitated by reading and re-reading the text of responses to open ended question for a full familiarisation**

• Though it’s a validation study it lacks statistical rigor in terms of validation

**Response: Thank you for the insightful and important comment. The current study had no intention of testing the psychometric properties of a scale. Instead, it focused on developing a KAP survey that is culturally suitable for the targeted multinational population of Qatar. The objective was to validate the alignment of the study tool with Qatari dietary guidelines. Thanks to your previous suggestion of clearly highlighting the study question in the introduction component this reads more clearly in the manuscript now.**

The most important question is why the author has chosen this particular method of development validation, are these best practices, if yes please quote references of the same

**Response: Reply: Thank you for the insightful comment. The WHO established principles of a KAP survey design were followed in choosing the current method (ref b). Relying on CVR and the panel of experts for developing the tools was described in ref c.**

**b. Guidelines for Assessing Nutrition-related Knowledge, Attitudes and Practices. Rome: Food and Agriculture; 2014 (http://www.fao.org/3/i3545e/i3545e05.pdf, accessed on 07/07/2024)**

**c. Hiew CC, Chin YS, Chan YM, Mohd Nasir MT (2015) Development and Validation of Knowledge, Attitude and Practice on Healthy Lifestyle Questionnaire (KAP-HLQ) for Malaysian Adolescents. J Nutr Health Sci 2(4): 407. doi: 10.15744/2393-9060.2.407 Volume 2 | Issue 4 Journal of Nutrition and Health Sciences**
Results

• Focus on Content Validity: The results section primarily focuses on demographics and completion time. Emphasize the percentage of items with acceptable CVR (e.g., >0.62) to demonstrate content validity.

**Response: Thank you for your important and insightful point. This information is highlighted in track changes as kindly suggested both in methods and result section of the manuscript.**

**‘Analysis of CVR values of the original version of the questionnaire form showed that more than 50% of questions had a CVR value of 1 (complete agreement). Another 26% had a value of 0.85 and 16.5% had a CVR of 0.69. Smaller CVR values were obtained in 4 questions only (around 5%)’.**

**Where:**

**CVR_i_ = value for an item on the test**

**n_e_ = number of experts indicating that an item is essential**

**N = total number of experts in the panel**

**Content validity ratio values (CVR)**

**Analysis of grading of the 73 questionnaire items (complete questionnaire included as supplementary document) included by the panel of experts (n=13) demonstrated that more than half of questions (52.1%) have a CVR value of which is followed by 26% with a value of 0.85 as depicted in table 2.**

Table 2: Frequency distribution of the CVR (Content Validity Ratio) of the 73 questionnaire items.

| **CVR Value** | **N** | **%** |
| --- | --- | --- |
| 1 | 38 | 52.1 |
| 0.7-0.92 | 19 | 26 |
| 0.62-0.69 | 12 | 16.4 |
| <0.62 | 4 | 5.5 |
| **Total** | **73** | **100** |

• Pilot Testing Results: Provide more details on the feedback from service users beyond percentages. Briefly mention specific comments on clarity, relevance, and length of the survey.

**Response: Thanks to your important suggestion we did thematic analysis of the responses of the open-ended question about feedback of the service users which is now described additionally in results section and depicted in table 6. We are grateful to your kind suggestion has it has further strengthened the results section of the study and highlights qualitative information from service users’ perspective.**

**Findings of the thematic analysis of the open-ended question pertaining to service users’ perceptions pertaining to the KAPs feasibility survey**

**Thematic analysis of the open-ended question about overall perceptions of the service users pertaining to the KAPS tool identified 3 important themes which were mainly i) clarity & readability of the questions ii) relevance of the instrument and iii) factors influencing service users’ participation in the survey. The main themes with subsequent sub-themes and related quotes are depicted in table 6.**

i) **Clarity & readability of the questions**

**The service users were overall satisfied with the clarity of the questions included in the KAPs feasibility survey. However, it was highlighted that the level of education and overall awareness about the general well-being can affect the interpretation and the clarity of the questions.**

**Similarly, a male Qatari service user commented:**

**The questionnaire reads well overall but not all parts can be the same. There must be few questions which might seem difficult to some. It is easy survey to complete though’.**

ii) **Relevance of the survey instrument**

**Regarding the relevance of the survey instrument the services users demonstrated the important of the cultural context and relatability of the tool. The services users documented the survey to be relevant and to truly captured what it claimed.**

**For example, an expat service user quoted relating to the significance of cultural context of the survey:**

**‘Every culture is different, how people live eat and maintain their health. A good tool must be made keeping this in mind’.**

iii) **Factors influencing service users’ participation in the survey**

**The services users highlighted the issue with the overall length of the survey and acknowledge that it can be influenced by individual patients’ preferences due to their daily life commitments and schedules during their healthcare visits as shown in table 6.**

**Table 6 Thematic analysis of perceptions of services users pertaining to the KAPS tool.**

| **Theme** | **Sub-theme** | **Quotes** |
| --- | --- | --- |
| **Clarity & readability of the questions** | **Variance in various questions** | **‘The questionnaire reads well overall but not all parts can be the same. There must be few questions which might seem difficult to some. It is easy survey to complete though’.** |
|  | **Level of understanding** | **‘Different people have different levels of education and basic understanding about their health. It can affect their interpretation and for some it can very clear what the question is asking but might be not for some’.** |
| **Relevance of the survey instrument** | **Capturing what it claims** | **‘I personally feel the questions in the survey can collect the data about my health and lifestyle choice that influence them. The tool seems to be true to what is made for, in delivering the information it claims to’.** |
|  | **Relatability to lifestyles within local community** | **‘After completing the survey, I feel that it did cover my lifestyle which can affect my health. I think it does relate to the general population in Qatar.’** |
|  | **Cultural context** | **‘Every culture is different, how people live eat and maintain their health. A good tool must be made keeping this in mind’.** |
| **Factors influencing service users participation in the survey** | **Length of the tool** | **‘I am usually a very busy person and if I have little time while visiting my doctor, I will not be that keen to fill the survey as I feel it’s a bit long and requires time to complete about my lifestyle and other health information.’**  **‘It doesn’t bother me much because I realize if I fill it this can benefit my health and the community. But I feel it will be different for different patients. How they feel as the survey has a lot of questions, for me I don’t mind doing it’.** |

• Data on Unfavorable Ratings: If the expert panel provided reasons for unfavorable ratings on CVR (irrelevant, difficult, etc.), consider mentioning the frequency of these reasons.

**Response: Thank you for the insightful comment. This information is highlighted in track changes as kindly suggested.**

**Questions that received unfavorable rating and labeled as "not necessary" as a contributor to the overall scale measure of a specific construct by two of the panelists were further examined for the reason of using that label. If the reasons were "irrelevant to the construct tested", or "Repeated elsewhere" this question was deleted. Reporting the reason as being "too technical and difficult for the audience," in addition to the two previously reported ones by any panelist requires amendments to that question. The second round of the panelist validation exercise considered 73 questions for assessment (the tool attached as supplementary document). The results are reported in table 2.**

• Combine Findings: The results section currently describes service user feedback in separate sections for healthy diet and physical activity. Consider combining these sections for improved readability.

**Response: This is a very useful point and thank you for highlighting it. We have combined the sections as kindly suggested (changes are highlighted in track changes).**

**Table 5 Describing the results of KAP feasibility survey on a sample of 11 service users.**

| **Positive feedback (Total N=11)** | **N** | **%** |
| --- | --- | --- |
| **Relevance of healthy diet section** |  |  |
| Thought that the questions about the Healthy Diet knowledge section are relevant to measuring knowledge about healthy food and eating? | 10 | 90.9 |
| Thought that the questions about the Healthy Diet attitudes section are relevant to measuring attitudes towards healthy food and eating? | 11 | 100.0 |
| Thought that the questions about the Healthy Diet practice section are relevant to measuring healthy food and eating behaviors? | 11 | 100.0 |
|  |  |  |
| **Relevance of physical activity section** |  |  |
| Thought that the questions about the Physical activity knowledge section are relevant to measuring knowledge about the role of active lifestyle and healthy weight? | 11 | 100.0 |
| Thought that the questions about the Physical activity attitude section are relevant to measuring attitudes towards active lifestyle and healthy weight? | 11 | 100.0 |
| Thought that the questions about the Physical activity practice section are relevant to measuring the physical activity practice? | 11 | 100.0 |

Discussion

• Connect Findings to Existing Literature: While you mention the importance of completeness in KAP surveys (citations provided), elaborate on how your study addresses this considering the length concerns raised by participants.

**Response: Thank you for a very important point. We totally agree with your suggestion. We have now added this in the discussion component highlighted in track changes in the discussion component.**

• Address Length Concerns: Acknowledge the trade-off between completeness and length. Discuss potential strategies to shorten the survey while maintaining validity, such as branching logic or creating different versions for specific contexts.

**Response: Thank you for a very important point. We have now acknowledged the trade-off between completeness and length. As kindly suggested, we have also mentioned the significance of exploring potential strategies to shorten the survey while maintaining validity, such as branching logic or creating different versions for specific contexts. Your point was insightful and helpful. This is specifically mentioned in conclusion section and abstract (highlighted in track changes).**

• Generalizability vs. Specificity: The discussion acknowledges limitations in generalizability due to the region-specific nature of the tool. However, emphasize the strengths of the tool for capturing healthy lifestyle knowledge in Qatar's primary care setting.

**Response: Thank you for the important suggestion. We have included this as a strength (highlighted as track changes). Many thanks for your invaluable input.**

**Reviewer #2:** 1. KAP was created by making some adjustments to the culture of the local community. What customs in the local community are unique and known to increase the risk of NCDs? Is there any previous research on this?

**Response: Thank you for highlighting a very important point. Evidence shows that Qatar has one of the highest prevalence of diabetes worldwide and a high proportion of people visiting non-communicable disease clinics in primary care settings have diabetes.(6, 7) Notably, 86% of patients diagnosed with diabetes in Qatar are uncontrolled, which will lead to an assumption that 12% of the entire population would be patients with uncontrolled diabetes (defined as patients having glycosylated hemoglobin Hb1Ac levels greater than 7% for the past 3 months) (8, 9). Studies report that medication non-adherence, variations in diverse socio-demographic and cultural characteristics within population sub-groups and lack of awareness of the disease associated complications among patients are some of the main factors for high uncontrolled diabetes prevalence.(8, 10)**

**Moreover, studies indicate a high prevalence non-communicable disease in the region which is closely associated with dietary habits and sedentary lifestyles among the local population.(11-13)**

2. is there a particular reason for involving respondents who are all individuals with a professional background in the health sector? why did the panelists not include people outside the health profession?

**Response: Thank you for your insightful comment. We have now also included service users’ perspectives in the results section (during the pilot testing of the tool) depicted in table 6 as kindly suggested.**

**The feedback was received particularly by panel of experts with a professional background pertaining for the inclusion of the relevant domains and items within the tool during the initial phase due to their experience and in-depth understanding of patient care and health service delivery mechanisms operated within primary care settings in the state of Qatar.**
3. The KAP is intended to be completed independently by patients, with input from panelists who consider the KAP too long, is the KAP reliable for use in the elderly population?

**Response: Thank you for highlighting a very important point. We have now acknowledged this as a limitation in the discussion component of the study (highlighted in track changes).**

4. Panelists' characteristics other than professional backgrounds were not explained. Age characteristics may affect their perception of the length or shortness of the questionnaire.

**Response: Thank you for the insightful comment. We have now included the demographic (gender and nationality in context to the WHO region) details in table 1. Age was not documented, and we agree with your point that it may affect somewhat of their perception of the length of the questionnaire. However, the age of retirement within PHCC is 60 years of age and most panelists were in the age range of 35-55 years of age. This is also highlighted as a limitation as kindly suggested.**

5. What type of questions were used? Can you explain a bit about it, for example, Likert scale, multiple choice, or open question?

**Response: Thank you for the insightful comment. Most of the questions measuring knowledge were measured on a dichotomous scale (yes, no, with an escape answer of I don’t know). The attitude questions were measured on a 5 grade LIKERT scale ranging from Strongly disagree to Strongly agree (Strongly Agree, Agree, Neutral, Disagree, strongly disagree). Other questions had choices to select the most suitable answer choice from. In the pilot testing of the tool there was a open ended question as well to capture general perceptions of the service users which is thematically analyzed and depicted in table 6.**

**6. It has not been explained which part of the KAP tool makes it too long and takes a long time to fill in.**

**Response: Thank you for the insightful comment. We have know included qualitative findings (themes) as depicted in table 6 which provide useful insights. We thank you for this suggestion as it has further strengthened the context and findings of the study.**

1.	Miles MB, Huberman AM. Qualitative data analysis: An expanded sourcebook: sage; 1994.

2.	Suter B. Tales to transit: Sub-Saharan African migrants’ experiences in Istanbul: Linköping University Electronic Press; 2012.

3.	Malterud K. Systematic text condensation: a strategy for qualitative analysis. Scandinavian journal of public health. 2012;40(8):795-805.

4.	Holloway I. Qualitative research in health care: McGraw-Hill Education (UK); 2005.

5.	Seale C, Gobo G, Gubrium JF, Silverman D. Qualitative research practice: Sage; 2004.

6.	Jaam M, Ibrahim MIM, Kheir N, Hadi MA, Diab MI, Awaisu A. Assessing prevalence of and barriers to medication adherence in patients with uncontrolled diabetes attending primary healthcare clinics in Qatar. Primary care diabetes. 2018;12(2):116-25.

7.	Zidan A, Awaisu A, El-Hajj MS, Al-Abdulla SA, Figueroa DCR, Kheir N. Medication-related burden among patients with chronic disease conditions: perspectives of patients attending non-communicable disease clinics in a primary healthcare setting in Qatar. Pharmacy. 2018;6(3):85.

8.	Jaam MJ. Barriers to medication adherence in patients with uncontrolled diabetes in a primary healthcare setting in Qatar: a mixed method triangulation study: Qatar University (Qatar); 2017.

9.	Gil J, Sicras-Mainar A, Zucchelli E. Uncontrolled diabetes and health care utilisation: panel data evidence from Spain. The European Journal of Health Economics. 2018;19:785-95.

10.	Jaam M, Hadi MA, Kheir N, Mohamed Ibrahim MI, Diab MI, Al-Abdulla SA, et al. A qualitative exploration of barriers to medication adherence among patients with uncontrolled diabetes in Qatar: integrating perspectives of patients and health care providers. Patient preference and adherence. 2018:2205-16.

11.	Syed MA, Alnuaimi AS, Zainel AJ, AA H. Prevalence of non-communicable diseases by age, gender and nationality in publicly funded primary care settings in Qatar. BMJ nutrition, prevention & health. 2019;2(1):20.

12.	Ng SW, Zaghloul S, Ali H, Harrison G, Popkin BM. The prevalence and trends of overweight, obesity and nutrition‐related non‐communicable diseases in the Arabian Gulf States. Obesity reviews. 2011;12(1):1-13.

13.	Elmusharaf K, Stanton R, Chestnov R. The case for investment in prevention and control of non-communicable diseases in Qatar. Geneva: UNDP, WHO, UNIATF, GHC. 2021.
