# Supplementary material for: Cultural & region-specific adaptation of KAP (Knowledge, attitude, and practice) tool to capture healthy lifestyle within primary care settings
Source: PLoS One. 2024 Dec 19;19(12):e0312852. doi: 10.1371/journal.pone.0312852 (PMC11658580; doi:10.1371/journal.pone.0312852)
Supplement: Attachment — Submitted filename: Reponse to reviewer comments.docx [file pone.0312852.s005.docx]

**To: Academic Editor PLOS ONE**

Dear **Jenny Wilkinson**,

We would like to thank you and the reviewers for providing further feedback on our submitted manuscript, " Cultural & region-specific adaptation of KAP (Knowledge, attitude, and practice) tool to capture healthy lifestyle within primary care settings".

We have carefully reviewed the feedback and made a thorough effort to address all the reviewers' comments. We believe these revisions have further strengthened the manuscript and addressed most of the reviewers' concerns. We have particularly elaborated and added details in the methodology and result section and revised the study topic as kindly suggested.

We have now submitted a revised manuscript with tracked changes for your review as instructed. We hope that the revisions meet with your approval and look forward for a positive response.

Thank you again for your kind support.

Sincere Regards

Dr Syed M Abbas

MBChB MPH MSc MRCGP MCPS HPE MS PhD

**Response to reviewer comments**

Comment: Your responses and manuscript revisions have been reviewed by the original reviewers and both reviewers have highlighted that while the work is of interest it lacks methodological and analytical rigor. Having reviewed their original and subsequent comments, and your revisions, I invite you to provide future revisions to address their concerns. Please focus on the clarity of the purpose of this study (is it about development of a KAP instrument or the modification of existing tools for a new audience) and the methodological rigor of the study, ensuring all relevant details are provided (e.g. how many individuals were interviewed, on what basis was n=11 deemed valid for the pilot study etc.). I also request that you consider the title of your work and whether this may be modified to improve clarity and alignment with your study.

**Response:** Thank you for the feedback, Jenny. We have carefully considered the points that you have mentioned and reviewer comments whilst submitting the revised version of the manuscript. We have modified the title of the study to provide a clear indication what they study has to offer. We have added more details in the methodology section pertaining to cognitive interviews. We have also highlighted the analysis details of the quantitative and qualitative data that was collected during the pilot testing phase of the study. The study highlights both the expert panel feedback (CVR) and pilot testing of the KAPs survey to capture service users perspective/feedback (both quantitatively and qualitatively). We agree that the pilot testing sample is not high. However, cognitive interviews (n=14) and quantitative & qualitative data was collected from service users during the pilot testing (n=11) of phase of the study provide a comprehensive perspective of the cultural and region-specific adaptation of the tool both from healthcare professionals and service users. Moreover, the tools are also adapted with expert panel feedback (n=13) in the early steps of the study.

**Response to reviewer 1 &2 comments**

**Reviewer #1:**

1) The language of the manuscript needs to be refined for redundancies and syntax errors. Some paragraphs in the introduction can be merged.

Response: Thank you for your comment. We have re-read the manuscript and merged paragraphs in the introduction section as kindly advised. Moreover, we have reviewed the manuscript to correct redundancies or any syntax errors. Thank you for suggesting.

2) The title "Designing a KAP (Knowledge, attitude, and practice) tool to capture healthy lifestyle within primary care", should be rewritten to familiarize the reader upfront with what is being done in this study. The word 'designing' is too vague, and connotates with the development of a tool. As I read it, the manuscript is concerned not with the designing of KAP questionnaire but aligning and modifying it with the cultural specifications. This should be hinted at in the title.

Response: Thank you for your kind suggestion. As advised, we have revised the topic of the study and now it reads as, ‘Cultural & region-specific adaptation of KAP (Knowledge, attitude, and practice) tool to capture healthy lifestyle within primary care settings".

3) The methodology is not sufficiently rigorous.

Response: Thank you for the comment. As kindly suggested, we have added details in the cognitive interviews, describing each step. We have now added details of analysis of both the quantitative and qualitative data that was collected during the pilot phase in this methodology study.

5) The pilot testing sample is too low.

Response: Thank you for the comment. We agree that the pilot testing sample is not high. However, cognitive interviews (n=14) and quantitative & qualitative data was collected from service users during the pilot testing (n=11) of phase of the study provide a comprehensive perspective of the cultural and region-specific adaptation of the tool both from healthcare professionals and service users. Moreover, the tools are also adapted with expert panel feedback (n=13) in the early steps of the study.

6) Elaborate more on what exactly was done in the cognitive interview, specifying if the interview was structured or unstructured. If the interview was structured, then what steps were taken.

Response: Thank you for the suggestion. We have now provided details in the methods section (highlighted as track changes) of each step of the cognitive interviews as well as data analysis of the qualitative data analyzed during the pilot testing of the KAPs survey.

*** Cognitive Interviews:** Cognitive interviews were conducted with a subset of participants to explore how they interpreted and responded to the modified survey items(1, 2). The interviews were conducted utilizing unstructured questions. A total of 15 participants were recruited for the cognitive interviews. The demographic details of the participants that participated in the cognitive interviews is shown in table 1. The interviews were conducted individually among participants. This step was included to further identify any underlying misconceptions or confusion pertaining to the culturally adapted and region specific KAP tool. The cognitive interview data was analyzed by synthesis and reduction which is a iterative process(3, 4). This involved the following steps(5):

i) Generating interview text: This initial step was implemented to generate interview text by collecting narratives from the respondents.

ii) Producing detailed summaries: This step involved synthesis of the interviews into summaries to further produce detailed summaries. The main aim of this step was to describe how and why each participants interpreted the questions in the KAPs survey, and the thought process involved in formulating the responses in context to their experiences with the services and interpretation of health outcomes highlighting the difficulties and challenges in generating these responses.

iii) Producing thematic schema: This step involved identifying and mapping common themes pertaining to the detailed phenomena captured and the processes involved in generating a response.

iv) Advanced schema: This step involved constructing advanced schema by comparing advanced themes across subgroups.

v) Drawing conclusions: The last step involved determining and explaining the performance of a question and its function in context to the socio-cultural background of the participants and their experiences

**Table 1 Demographic details of participants for cognitive interviews (n=15)**

| **Profession/ status** | **n** | **Age** | **Gender** | **Ethnicity** | **Years of experience/ accessibility to primary health services** |
| --- | --- | --- | --- | --- | --- |
| Family Medicine Physicians | 4 | 35- 45 years of age | 3, Male, 1 Female | 2, Europe, 2 Eastern Mediterranean | Between 5-10 years |
| Dieticians | 4 | 25-34 years of age | 3, Female, 1 Male | 1, Africa, 2 Eastern Mediterranean, 1, Europe | Less than 5 years |
| Public Health Officers | 3 | 25-34 years of age | 2, Males, 1 Female | 3, Eastern Mediterranean | Between 5-10 years |
| Service users of PHCC | 4 | 18-65 years of age | 2, Male, 2 Female | 3, Eastern Mediterranean, 1, East Asia | Between 5-10 years |

7) CVI ratings can be disclosed in the result section of the abstract, if feasible.

Response: Thank you for the important point. We have now included CVI rating in the results section of the abstract as kindly suggested. We have also highlighted the main themes that resulted from the cognitive interviews in the results section of the abstract.

**Reviewer #2**

 Thank you for responding to the review, this study is interesting enough to assess KAP (Knowledge, attitude, and practice), so it is expected to capture a healthy lifestyle in primary care. This study certainly raises important issues that can be used for the local community in Qatar. However, this manuscript does not report well enough on how the development of these tools can fit these interests. Stronger arguments need to be made about the uniqueness of the local community about the NCDS so that these tools are necessary or can help address the problems.

Response: We have carefully considered the points that you have mentioned and reviewer comments whilst submitting the revised version of the manuscript. We have modified the title of the study to provide a clear indication what they study has to offer. We have also included the points that you have highlighted in the introduction and discussion component.

Research methods related to building instruments need to be strengthened. It is necessary to report with clear stages when research is done with mixed methods and appropriate analysis for Likert scales.

Response: As kindly suggested, we have added details in the cognitive interviews, describing each step. Moreover, details are highlighted in red of the analysis steps of both the quantitative and qualitative data that was collected in this methodology study. The methods section details 9 steps in total overall constituting more than 1000 words of the manuscript.

But in general, this manuscript is fascinating. Hopefully, it can be improved further. Good luck.

Response: Thank you for your kind words and encouragement. It means a lot.

1.	Bekerian D, Dennett J. The cognitive interview technique: Reviving the issues. Applied Cognitive Psychology. 1993;7(4):275-97.

2.	Memon A, Gawrylowicz J. The cognitive interview. The handbook of communication skills. 2018:511-30.

3.	Miles MB, Huberman AM. Qualitative data analysis: An expanded sourcebook: sage; 1994.

4.	Suter B. Tales to transit: Sub-Saharan African migrants’ experiences in Istanbul: Linköping University Electronic Press; 2012.

5.	Malterud K. Systematic text condensation: a strategy for qualitative analysis. Scandinavian journal of public health. 2012;40(8):795-805.
